# Disease Clearance in Ulcerative Colitis: A Narrative Review

**DOI:** 10.1002/ueg2.12714

**Published:** 2025-04-16

**Authors:** Silvio Danese, Laurent Peyrin‐Biroulet, Vipul Jairath, Ferdinando D'Amico, Shashi Adsul, Christian Agboton, Fernando Magro

**Affiliations:** ^1^ Department of Gastroenterology and Endoscopy IRCCS San Raffaele Hospital and Vita‐Salute San Raffaele University Milan Italy; ^2^ Department of Gastroenterology INFINY Institute INSERM NGERE Vandœuvre‐lès‐Nancy France; ^3^ Department of Medicine Division of Gastroenterology Western University London Canada; ^4^ Alimentiv London Canada; ^5^ Takeda Cambridge Massachusetts USA; ^6^ Center for Health Technology and Services Research (CINTESIS) Faculty of Medicine of the University of Porto Porto Portugal

**Keywords:** course, disease modification, histological healing, mucosal healing, progression, remission, STRIDE, target, therapy, vedolizumab

## Abstract

Ulcerative colitis (UC) is a chronic relapsing disease with significant associated risks such as colectomy, hospitalization, or colorectal cancer. A treat‐to‐target approach that mitigates disease activity and progression from an early stage is needed. The latest STRIDE II guidelines advocate for clinical and endoscopic remission as the main therapeutic targets in the management of UC; however, histological remission is increasingly being recognized as an important outcome. The concept of disease clearance, a composite outcome comprising clinical, endoscopic, and histological remission, has been proposed as a potential target for patients with UC and has been precisely defined by the International Organization for the Study of Inflammatory Bowel Disease, with the aim of standardizing its use in clinical practice and research. Despite challenges, including variable standardized definitions and uncertainties regarding the timing of reaching different definitions of remission, disease clearance corresponds to comprehensive disease control, and its use as an outcome could help clinicians to better evaluate the actual status of the disease. Furthermore, achieving disease clearance may be related to an improved disease course, positive long‐term outcomes, and an improvement in health‐related quality of life. Real‐world evidence supports the feasibility of achieving disease clearance with various treatment modalities, including vedolizumab, the only gut‐selective antilymphocyte trafficking drug. The aim of this narrative review is to explore the concept of disease clearance in patients with disease clearance, mainly focusing on trials evaluating vedolizumab but also other biologics.

## Graphical abstract

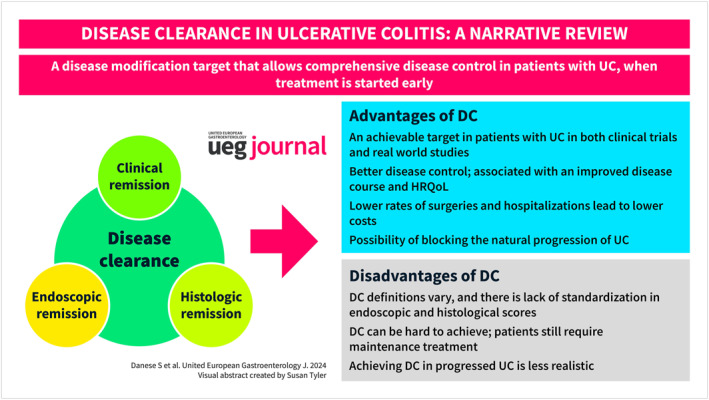

## 1 Introduction

Ulcerative colitis (UC) presents as a lifelong and progressive disease, characterized by chronic relapsing and remitting conditions [1]. Consequently, effective management strategies addressing both short‐ and long‐term outcomes are needed. Colectomy, hospitalization, and the risk of colorectal cancer are the major concerns in the management of UC [2]. Intestinal fibrosis, a common complication in UC, is characterized by a thickening of the muscularis mucosa and excessive extracellular matrix deposition in the submucosa due to chronic inflammatory and reparative responses [3, 4].

To achieve better outcomes for patients with UC, a treat‐to‐target approach aiming to reduce bowel dysfunction by mitigating disease activity at its earliest stage is needed to effectively limit both disease progression and inflammation. The STRIDE II guidelines recommend using clinical and endoscopic remission as the main therapeutic targets in the management of UC [5]. Clinical remission is considered an intermediate treatment target and should be defined using patient‐reported outcomes‐2 or a partial Mayo score (Table 1) [5]. Whereas the Mayo score, which is the most used index for evaluation of disease activity and severity, includes stool frequency, rectal bleeding, physician's global assessment, and an endoscopic subscore ranging from 0 to 12 points, the partial Mayo score does not include endoscopic assessment [6].

**TABLE 1:** Definitions of remission.

	Clinical	Endoscopic	Histological
Treatment target type	Intermediate	Long term	Long term
Definition	PRO2 (rectal bleeding subscore of 0, and stool frequency subscore of 0) Partial mayo score (< 3 and no score > 1)^a^	MES of < 1 UCEIS score of ≤ 1 point HEMI = MES of ≤ 1 + GS of 3.1 HEMR = MES of 0 + GS of 2.0 or ≤ 2B = 0^c^	GS of ≤ 6 (continuous) GS of ≤ 2B.0 (categorical) NI grade ≤ 1 RHI score of ≤ 3^b^

Abbreviations: GS, Geboes Score; HEMI, histologic endoscopic mucosal improvement; HEMR, histologic endoscopic mucosal remission; MES, Mayo endoscopic score; NI, Nancy Index; PRO2, patient‐reported outcomes‐2; RHI, Robarts Histopathology Index; UCEIS, Ulcerative Colitis Endoscopic Index of Severity.

^a^ The partial Mayo score does not include endoscopic assessment.

^b^ With subscores of 0 for lamina propria neutrophils and neutrophils in the epithelium and without ulcers or erosion.

^c^ Category 2B refers to no increase in neutrophils in the lamina propria.

Endoscopic remission is considered a long‐term treatment target and should be defined as a Mayo endoscopic score (MES) of < 1 or Ulcerative Colitis Endoscopic Index of Severity of ≤ 1 point (Table 1) [7]. The MES is the most extensively used endoscopic index in clinical practice and trials because it is easy and practical, conveys good predictive value, and provides a simple visual representation of the degree of endoscopic inflammation in UC (with scores ranging from 0 to 3) [6].

Although the STRIDE II guidelines indicate that histological remission is currently not a formal target, it can be used as an adjunct to endoscopic remission to represent a deeper level of healing [5]. A significant proportion of patients with endoscopic remission experienced histologic disease activity (20%–30%). Histological remission is being increasingly recognized as a therapeutic goal in patients with UC [8, 9], with accumulating evidence indicating that microscopic activity persists in endoscopically quiescent UC, that there is a time lag between histological changes and clinical remission after treatment, and that the absence of histological activity can predict lower relapse rates, as well as lower rates of hospitalizations, surgeries, and subsequent neoplasias. Measures used to assess histological activity include the Geboes Score (GS), the Nancy Index (NI), or the Robarts Histopathology Index (RHI) (Table 1) [2]. The GS measures structural changes, chronic inflammation, the presence of neutrophils in the epithelium and lamina propria, crypt disruption, erosion, and ulcers, on a scale from 0 to 5.4, with higher scores indicating more severe disease activity [2, 10]. NI evaluates ulceration, acute inflammatory infiltrate, and chronic inflammatory infiltrate [2]. The RHI measures the presence of neutrophils in the lamina propria and mucosa, ulcerations, or erosion together with the chronic inflammatory infiltrate on a scale of 0–33, with higher scores indicating more severe disease activity [2, 10]. Using these tools, histological response is defined as a continuous GS of ≤ 12, score of < 3.0 in GS, RHI score of ≤ 9, or NI grade ≤ 1, whereas histological remission is defined as a continuous GS of ≤ 6, score of ≤ 2B.0 in GS, NI grade ≤ 1, or RHI score of ≤ 3 (Table 1) [11].

Clinical symptom scores have been shown to correlate well with endoscopic scores; however, some patients with endoscopic scores of 0–1 still exhibit symptoms, and clinical symptoms alone cannot be relied upon to assess disease activity [12, 13]. In addition, the presence of histological inflammation may, in part, explain the persistence of symptoms in patients with endoscopic healing [12, 13]. Therefore, the combination of clinical, endoscopic, and histological activity endpoints may allow the achievement of disease control. Two endpoints that combine both endoscopic and histologic disease activity in clinical trials are histologic endoscopic mucosal improvement (HEMI) and histologic endoscopic mucosal remission (HEMR) (Table 1) [14]. Early achievement of HEMI at the end of induction therapy has been shown to be a better predictor of clinical and symptomatic outcomes at the end of maintenance therapy than histologic improvement or endoscopic remission alone [14]. Early achievement of HEMR was associated with a numerically greater likelihood of improvement in clinical outcomes at the end of maintenance therapy compared with achievement of HEMI without HEMR [15]. Furthermore, the incremental prognostic value of histologic remission in patients with UC in clinical and endoscopic remission has been demonstrated in several studies [16, 17]. It was reported that patients with endoscopic remission but persistent histological activity have a 63% higher risk of 12‐month clinical relapse than those who achieve histological remission [18].

With the expansion of treatments targeting the underlying pathophysiological processes of UC, there is an opportunity to develop management strategies to improve both short‐term (clinical remission) and long‐term (endoscopic remission) disease control as well as to enhance histological outcomes [19, 20]. Treatment options should account for clinical, endoscopic, and histologic outcomes, as none of these three components taken separately is sufficient to ensure optimal results for patients, and discrepancies exist between them [13]. In particular, histological outcomes need to be considered, as they have been shown to be associated with positive prognosis of long‐term outcomes [13]. Indeed, the VARSITY trial (NCT02497469), the first head‐to‐head comparison of biologics in inflammatory bowel disease (IBD), compared histological outcomes in patients treated with either vedolizumab or adalimumab [21]. The results showed that at 52 weeks, a significantly higher number of patients treated with vedolizumab achieved histologic remission (assessed using both GS and RHI) compared with adalimumab [21]. A post hoc analysis from VARSITY also showed that vedolizumab‐treated patients achieved significantly higher rates of histologic remission (RHI score of ≤ 2) plus endoscopic improvement (MES of ≤ 1) than adalimumab‐treated patients at 52 weeks (30.5% vs. 14.5%, respectively) [21].

An expert consensus published by the International Organization for the Study of Inflammatory Bowel Disease (IOIBD) has proposed a definition for disease clearance (DC) as an outcome in patients with UC, and stated that DC is achievable with current treatments for IBD [22]. This definition may allow for standardization of DC evaluation [8]. In the current article, we review the concept of DC in patients with UC, with a focus on vedolizumab studies. Vedolizumab was selected because of its distinctive mechanism of action, which prevents lymphocytes entering the mucosa by reducing the proportion of α_4_β_7_‐expressing CD4+ and CD8+ T cells [23]. Moreover, mucosal eosinophil abundance has been shown to be associated with a response to vedolizumab [24].

## 2 IOIBD Definition of DC

**FIGURE 1:**
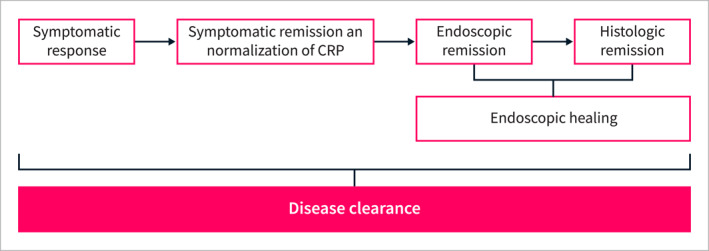
Disease clearance in ulcerative colitis. CRP, C‐reactive protein.

The IOIBD defined DC as deep and comprehensive remission including clinical remission assessed with a partial Mayo score of 0, endoscopic remission assessed with an MES of 0, and histologic remission assessed with an NI grade of 0 (Figure 1) [22]. Achieving these components is believed by many physicians to decrease the progression of UC in the long term and prevent associated complications [25]. The Selecting end PoInts for disease‐modIfication Trials (SPIRIT) consensus recommends that the ultimate therapeutic goal in Crohn's disease (CD) and UC should be preventing the impact of the disease on patients' lives (health‐related quality of life [HRQoL], disability, fecal incontinence), midterm complications (encompass bowel damage in CD, IBD‐related surgery and hospitalizations, disease extension in UC, extraintestinal manifestations, permanent stoma, short bowel syndrome), and long‐term complications (gastrointestinal and extraintestinal dysplasia or cancer, mortality) [26]. We suggest that achieving DC is a method of comprehensive disease control that may be the first step toward disease modification in patients with UC, rather than simply being a quantitative tool for a deeper remission target.

## 3 Is a Score of 0 a Realistic Target or Are Less Stringent Target Goals Still Valid to Achieve DC?

The pursuit of DC often raises the question of whether achieving a score of 0 is a realistic target or if less stringent goals remain valid. Mucosal healing, often defined as an MES of ≤ 1, gains significance when complete endoscopic healing is considered (MES of 0), which is associated with better disease outcomes [5, 6]. Despite evidence supporting the achievability of DC, the proportion of patients attaining this outcome remains limited [8, 27]. Regardless of the drug used in clinical trials or retrospective data from international cohorts, the percentage of patients able to achieve DC remains low and DC is a high bar to achieve, with the potential for disease modification [25]. A multicenter retrospective study using data from Australian patients with UC revealed that 61% of patients achieved clinical remission, 35% were in both clinical and endoscopic remission, and only 16% were in combined clinical, endoscopic, and histological remission [28]. In that study, clinical remission was defined as the absence of rectal bleeding and normal stool frequency, endoscopic remission was defined as an MES of ≤ 1, and histological disease activity was defined (using the Truelove and Richards Index) as the absence of erosion, crypt abscesses, or neutrophilic inflammation (with or without architectural distortion) [28].

## 4 Challenges With Using DC as a Treatment Target in Patients With UC

The use of DC as a treatment target in patients with UC presents several challenges. One notable obstacle is the variability in the definitions of DC in clinical trials, making comparisons across trials challenging. Contrary to the traditional belief that histological remission follows clinical and endoscopic remission, patients have been shown to achieve histological remission simultaneously with endoscopic remission [29]. This variance in timing could be attributed to the specific mechanism of action of drugs, with those targeting the tissue potentially achieving histological remission earlier than systemically acting drugs. The feasibility of achieving DC may also depend on the progression of IBD. In patients with IBD that has already progressed, achieving DC becomes less realistic due to suboptimal medication response and potential irreversible damage [25]. Real‐world evidence supports this notion, indicating that patients achieving DC tend to have a shorter disease duration than those not achieving DC, possibly indicating a more challenging disease to treat in patients with longstanding IBD [30]. Moreover, the value of histologic activity as an independent risk factor for progressive disease in asymptomatic patients appears to be time limited, particularly within the first 36 months after biopsy [31].

Due to the need to balance the benefit‐risk profiles of treatments, physicians may exhibit reluctance to escalate treatment in patients who have achieved clinical remission but not endoscopic or histological remission [25]. This hesitation is partly rooted in the misconception that UC is less progressive than CD, which may cause hesitation in introducing more effective treatments earlier in the disease course [32]. The long‐term risks associated with immunosuppressive drugs should not outweigh the potential advantages of starting biological therapy early and preventing irreversible bowel injury. However, the current STRIDE II guidelines, which were developed for routine practice and not for clinical trials, do not explicitly incorporate histologic remission [5, 9]. This exclusion stems partly from the requirement for biopsy sampling, making histological remission less practical for routine clinical practice compared with clinical and endoscopic remission [6]. Indeed, an international survey of physicians treating patients with UC in real‐world practice has indicated that while most physicians consider histological activity important for UC management, the majority reported that standard indices were not used. In contrast, most physicians indicated using MES for endoscopic scoring [33].

## 5 Benefits of Using DC as a Treatment Target in Patients With UC

DC has emerged as a valuable treatment target in the management of UG, offering several advantages supported by real‐world studies and clinical trials. Real‐world studies underscore that achieving DC in patients with UC is associated with an improved disease course and a lower rate of negative outcomes such as hospitalization and surgery [22, 34]. DC is a particularly realistic goal when treatment is initiated early, at the time of diagnosis [25]. Current IBD treatments have demonstrated efficacy in achieving DC, as substantiated by evidence from both real‐world studies and clinical trials [22]. Despite this, it is important to emphasize that achieving DC does not equate to a cure for UC and that patients who achieve DC still need maintenance treatment, and may still experience disease relapse. However, embracing DC as a method of comprehensive disease control holds the potential to mitigate UC‐related complications and consequently reduce indirect health care costs [8]. Notably, the significantly lower rates of hospitalizations and surgeries in patients achieving DC translate into decreased health care costs associated with UC [30].

## 6 Evidence of DC From Real‐World Studies and Clinical Trials

The ability to achieve DC in patients with UC is well documented in both real‐world studies and clinical trials, shedding light on its clinical significance (Table 2). Real‐world studies report that DC is a feasible outcome with various treatment modalities, encompassing conventional therapies and biologics, and is associated with lower risks of negative outcomes, hospitalizations, and surgeries [30, 35, 36]. The phase 3b, randomized, double‐blind, double‐dummy, active‐control VARSITY trial compared vedolizumab and adalimumab as maintenance treatments in patients with moderately to severely active UC, and demonstrated that vedolizumab led to significantly higher rates of clinical remission (primary outcome) and endoscopic improvement, histologic remission, and minimal histologic disease (exploratory endpoints) compared with adalimumab at week 52 [10]. Clinical remission was observed in 31.3% of patients in the vedolizumab group compared with 22.5% in the adalimumab group; endoscopic improvement (defined as an MES of ≤ 1) was observed in 39.7% of patients treated with vedolizumab compared with 27.7% treated with adalimumab; histological remission (defined as a GS of < 2.0 and an RHI score of < 3) was observed in 10.4% of patients in the vedolizumab group compared with 3.1% in the adalimumab group; and minimal histologic disease (defined as a GS of < 3.2 and an RHI score of < 5) was observed in 33.4% and 13.7% of vedolizumab‐ and adalimumab‐treated patients, respectively. Histological remission and histological disease were also assessed using the RHI score and similar results were observed. A total of 37.6% of vedolizumab‐treated patients compared with 19.9% of adalimumab‐treated patients, and 42.3% of vedolizumab‐treated patients compared with 25.6% of adalimumab‐treated patients, respectively, reached histologic remission and histological disease. DC, defined as clinical remission (partial Mayo score of ≤ 2 and no individual subscore of > 1 [excluding sigmoidoscopy subscore]), endoscopic improvement (MES of ≤ 1), and absence of active histological disease (minimum histological disease activity: RHI score of < 5), was assessed as an endpoint in a subanalysis in this study [37]. Almost 30% of vedolizumab‐treated patients achieved DC compared with 16% of adalimumab‐treated patients. Finally, the absence of inflammatory burden at baseline was associated with DC at week 52 in both treatment groups.

**TABLE 2:** Summary of studies using DC as an outcome in patients with UC.

Study	Treatment	Definition of DC	Outcome
Real‐world studies			
Real‐world cohort study [35] (*N* = 79)	Treatments not specified	Clinical (partial mayo score of ≤ 2), endoscopic (MES of ≤ 1), and histological (NI grade 0) remission	• Patients with UC in clinical, endoscopic, and histological remission have a significantly lower risk of hospitalization, complications, and surgical intervention
Multicenter, retrospective, real‐world cohort study [30] (*N* = 494)	Multiple treatments, most commonly thiopurines, infliximab, adalimumab, and vedolizumab	Clinical (partial mayo score of ≤ 2, with no subscore), endoscopic (MES of 0), and histological (NI grade 0) remission	• 22.1% (109/494) of patients had DC after induction
			• There were significantly lower risks of any negative outcomes, hospitalizations, and surgeries in patients with versus without DC within 16 weeks of induction therapy
Retrospective, real‐world cohort study [36] (*N* = 56)	Aminosalicylates	Clinical (partial mayo score of ≤ 2), endoscopic (MES of ≤ 1), and histological remission (chronic inactive/quiescent colitis)	• DC in patients with UC was associated with a high cumulative probability of maintaining remission
Clinical trials			
Post hoc analysis from 4 Phase 3 clinical trials in patients with mild‐to‐moderate UC [27] (*N* = 860)	Mesalazine granules	Clinical (clinical activity index), endoscopic remission (local endoscopists using the endoscopic index), and histological analysis performed by central reading using the histological index	• 20.0% of patients with mild‐to‐moderate UC achieved DC with mesalazine granules post induction
VARSITY [37] (*N* = 769)	Vedolizumab versus adalimumab	Clinical remission (partial mayo score of ≤ 2 and no individual subscore of > 1 excluding sigmoidoscopy subscore), endoscopic improvement (MES of ≤ 1), and absence of active histologic disease (minimum histological disease activity: RHI score of < 5)	• DC was achieved in almost one‐third of patients in the vedolizumab treatment group
			• DC at week 52: Vedolizumab, 29.2%; adalimumab, 16.3%
			• Fewer patients with inflammatory burden at baseline achieved DC
VERDICT [50] (*N* = 331)	Vedolizumab	Symptomatic remission (mayo rectal bleeding subscore of 0), endoscopic remission (MES of ≤ 1), and histologic remission (GS of < 2.0)	• With 50% of the target population randomized, 65% had reached clinical remission, 39% had reached clinical and endoscopic remission, and 37% had reached DC at week 16
(VERDICT ECCO 2024) [39] (*N* = 553)	Vedolizumab	Symptomatic remission (mayo rectal bleeding subscore of 0), endoscopic remission (MES of ≤ 1), and histologic remission (GS of < 2.0)	• At week 16, 86/212 (41%) of patients achieved DC, including 77/186 (41%) biologic‐naïve patients and 9/26 (35%) biologic‐exposed patients
UNIFI (Phase 3 study) [40] (*N* = 961)	Ustekinumab versus placebo	Simultaneous achievement of symptomatic remission (i.e., mayo stool frequency subscore of < 1 and rectal bleeding subscore of 0) and HEMH (i.e., MES of < 1 and histologic improvement using GS‐based criteria: Absence of erosion or ulcerations, absence of crypt destruction, and < 5% of crypts with neutrophil infiltration)	• At week 8, the rate of DC was significantly higher with ustekinumab (130 and 6 mg/kg doses) than placebo
			• At week 44, a significantly higher proportion of patients treated with ustekinumab (90 mg) than placebo achieved DC
Post hoc analysis from ELEVATE UC 52 (*N* = 409) and ELEVATE UC 12 (*N* = 334)	Etrasimod versus placebo	DC was defined as NI grade 0, MES of 0, rectal bleeding subscore of 0, and stool frequency subscore of 0 or 1 (if 1, must have *a* ≥ 1‐point reduction from baseline)	• ELEVATE UC 52: At week 12, 23/274 (8.4%) etrasimod‐treated patients and 2/135 (1.5%) placebo‐treated patients achieved DC. At week 52, 51/274 (18.6%) etrasimod‐treated patients and 5/135 (3.7%) placebo‐treated patients achieved DC
			• ELEVATE UC 12: 22/222 (9.9%) etrasimod‐treated patients and 5/112 (4.5%) placebo‐treated patients achieved DC

Abbreviations: DC, disease clearance; GS, Geboes Score; HEMH, histo‐endoscopic mucosal healing; MES, Mayo endoscopic subscore; NI, Nancy Index; RHI, Robarts Histopathology Index; UC, ulcerative colitis.

VERDICT (actiVE ulcerative colitis, a RanDomized Controlled Trial; NCT04259138) is an ongoing, randomized, controlled trial designed to determine the optimal treatment target in moderately to severely active UC. In this study, patients are being randomized to 3 groups according to treatment target: group 1 (corticosteroid‐free [CSF] symptomatic remission [defined as a Mayo rectal bleeding subscore of 0]), group 2 (CSF symptomatic remission + endoscopic improvement [MES of ≤ 1]), and group 3 (CSF symptomatic remission + endoscopic improvement + histologic remission [GS of < 2B.0.]). This study aims to explore the superiority of a treatment target of CSF symptomatic plus endoscopic plus histologic remission over CSF symptomatic remission alone within an 80‐week follow‐up [38]. Therapy is being administered according to a treatment algorithm based on each patient's UC treatment at screening. Patients follow treatment algorithm that features early introduction of vedolizumab with no dose escalation before week 16 and no week 10 dosing. As of March 1, 2023, of the 432 patients who were enrolled and randomized (approximately 65% of the target population), 51% (95% confidence interval [CI]: 40%–61%), 37% ([95% CI: 29%–46%], and 33% [95% CI: 27%–40%]) met their treatment targets in groups 1, 2, and 3, respectively [38]. In addition, an interim analysis assessed the achievement of CSF DC at week 16 in group 3. At the time of this analysis (August 18, 2023), 553 patients were enrolled in VERDICT, with 253 patients assigned to target group 3, of whom 216 patients were biologically naïve and 37 patients were biologically exposed. Among the 212 patients with remission target status available, 86 (41%) achieved CSF DC remission, including 77 (41%) of the 186 biologic‐naïve patients and 9 (35%) of the 26 biologic‐exposed patients. In the intent‐to‐treat population (*n* = 253), CSF DC was achieved in 34% of patients, 36% of biologic‐naïve patients, and 24% of biologic‐exposed patients. All patients with DC received vedolizumab treatment from baseline [39].

UNIFI (NCT02407236) was a phase 3 study of ustekinumab versus placebo in patients with moderate‐to‐severe UC. A subanalysis of the UNIFI trial investigated the effects of induction and maintenance treatment with ustekinumab on DC [40]. DC was defined as the simultaneous achievement of symptomatic remission (Mayo stool frequency subscore of < 1 and rectal bleeding score of 0) and histo‐endoscopic mucosal healing (MES of < 1) and histological improvement using GS‐based criteria (absence of erosion or ulcerations, absence of crypt destruction, and < 5% crypts with neutrophil infiltration). After induction treatment, patients with moderate‐to‐severe UC who were treated with ustekinumab (130 mg and 6 mg/kg doses) had a significantly higher rate of DC at week 8 versus placebo. In addition, a significantly higher proportion of patients treated with ustekinumab (90 mg) rather than placebo achieved DC at week 44 of maintenance treatment. Achievement of early DC was associated with better long‐term outcomes (including clinical remission, histologic improvement, endoscopic improvement) and histo‐endoscopic mucosal healing at week 44.

Another post hoc analysis evaluated the feasibility of etrasimod, a sphingosine 1‐phosphate receptor 1, 4, 5 modulator, to achieve DC [41, 42]. This analysis of data from the ELEVATE UC 52 (NCT03945188) and ELEVATE UC 12 (NCT03996369) trials of etrasimod in patients with moderately to severely active UC evaluated the effect of etrasimod on DC at weeks 12 and 52. In this study, DC was defined as NI grade 0, endoscopic subscore of 0, rectal bleeding subscore of 0, and stool frequency subscore of 0 or 1 (if 1, must have *a* ≥ 1‐point reduction from baseline). The results showed that etrasimod was significantly better than placebo in achieving DC at weeks 12 and 52 [41, 42].

## 7 DC and Quality of Life

DC in patients with UC extends beyond clinical outcomes, with a significant impact on HRQoL, which has been suggested as a relevant endpoint for IBD management [6].

Clinical activities such as increased bowel frequency, urgency, and rectal bleeding have been identified as key factors negatively affecting HRQoL [22]. The Inflammatory Bowel Disease Questionnaire 32 and the Inflammatory Bowel Disease Questionnaire 36 are commonly employed disease‐specific tools in UC [22]. Achieving DC, an endpoint that combines clinical, endoscopic, and histological outcomes, may be associated with improved UC control and improved quality of life [22]. However, further research is warranted to delineate the degree to which DC affects HRQoL compared with clinical remission alone in patients with UC.

## 8 Treatment Strategies for Achieving DC in UC

Studies regarding the optimal treatments for achieving DC in patients with UC are limited. Current medications for IBD face challenges in achieving endoscopic healing, despite this being an earlier and more achievable target compared with histological healing [43]. In fact, there is a prevailing worry that adopting histological healing as a target might lead to rapid cycling through medications, potentially deeming the treatment a failure [43]. Notably, more efficacious early treatments may lead to better rates of histologic healing. For instance, an early top‐down approach may lead to better mucosal and histological healing [43].

Monoclonal antibodies such as infliximab (chimeric human antitumor necrosis factor‐α), adalimumab (completely humanized antitumor necrosis factor‐α), vedolizumab (anti‐integrin *α*_4_β_7_), and ustekinumab (interleukin‐12 and ‐23 antagonist) are indicated for use in patients with CD and UC [44]. Small molecules for oral administration, including JAK inhibitors (tofacitinib, upadacitinib) and S1P modulators (ozanimod) are also available. Additionally, the potential for dual‐targeted therapy in patients who had an incomplete response to monotherapy as an optimal strategy for managing IBD and achieving DC has been explored, with limited data assessing outcomes such as clinical remission, mucosal healing, or endoscopic response [45, 46, 47]. However, direct assessments of the dual‐targeted therapy impact on DC remain an area for further investigation, as are the safety profiles of using different biologics or small molecules in combination [46].

Lastly, for the treatment effects of different biologics to be compared, the definition of DC in UC will need to be standardized and validated. The current definition of DC may evolve to include other noninvasive markers of inflammation such as fecal calprotectin and intestinal ultrasound. Recent evidence indicates that a single measure of endoscopic remission using electronic virtual chromoendoscopy can accurately predict histologic remission [48, 49]. In the future, this technique could allow the simultaneous evaluation of endoscopy and histology.

## 9 Conclusions

DC is an important goal for the comprehensive treatment of patients with UC. The ongoing VERDICT trial in patients with moderate‐to‐severe UC will provide further validity for the use of DC as a treatment target. Further work is needed regarding the optimum treatments to achieve DC. It is important to emphasize that achieving DC does not equate to a cure and that patients who achieve DC still need maintenance treatment for their UC. It will be important to investigate risk factors associated with DC as a clinically useful means of identifying patients with the greatest chance of achieving DC. Finally, the notion of DC implies that there is a disease “trajectory,” so in order to make DC a reality in clinical practice, further studies are needed to characterize these trajectories.

## Author Contributions

All authors were involved in drafting or revising the manuscript critically for important intellectual content and approved the final submitted version.

## Conflicts of Interest

Silvio Danese reports receiving lecture fees from and serving as a consultant for AbbVie, Allergan, Biogen, Boehringer Ingelheim, Celgene, Celltrion, Ferring, Hospira, Johnson & Johnson, Merck, Merck Sharp & Dohme, Mundipharma, Pfizer, Sandoz, Takeda, TiGenix, UCB, and Vifor. Laurent Peyrin‐Biroulet reports receiving consulting fees from AbbVie, Abivax, Adacyte, Alimentiv, Amgen, Applied Molecular Transport, Arena, Banook, Biogen, Bristol Myers Squibb, Celltrion, Connect Biopharm, Cytoki Pharma, Enthera, Ferring, Fresenius Kabi, Galapagos, Genentech, Gilead, GlaxoSmithKline, Gossamer Bio, IAC Image Analysis, Index Pharmaceuticals, Inotrem, Janssen, Medac, Mopac, Morphic, Merck Sharp & Dohme, Nordic Pharma, Novartis, Oncodesign Precision Medicine, ONO Pharma, OSE Immunotherapeuthics, Pandion Therapeutics, Par' Immune, Pfizer, Prometheus, Protagonist, Roche, Samsung, Sandoz, Sanofi, Satisfay, Takeda, Telavant, Theravance, Thermo Fischer, TiGenix, Tillots, Vectivbio, Ventyx, Viatris, and Ysopia; grants from Celltrion, Fresenius Kabi, Medac, Merck Sharp & Dohme, and Takeda; and lecture fees from AbbVie, Amgen, Arena, Biogen, Celltrion, Eli Lilly and Company, Ferring, Galapagos, Genentech, Gilead, Janssen, Medac, Merck Sharp & Dohme, Nordic Pharma, Pfizer, Sandoz, Takeda, Tillots, and Viatris. Vipul Jairath reports receiving consulting/advisory board fees from AbbVie, Alimentiv, Arena, Asahi Kasei Pharma, Asieris, AstraZeneca, Bristol Myers Squibb, Celltrion, Eli Lilly and Company, Ferring, Flagship Pioneering, Fresenius Kabi, Galapagos, Genentech, Gilead, GlaxoSmithKline, Janssen, Merck, Mylan, Pandion, Pendopharm, Pfizer, Protagonist, Reistone Biopharma, Roche, Sandoz, Second Genome, Takeda, Teva, Topivert, Ventyx, and Vividion; and speaker fees from AbbVie, Ferring, Fresenius Kabi, Galapagos, Janssen, Pfizer, Shire, and Takeda. Ferdinando D'Amico serves as a speaker for Galapagos, Janssen, Omega Pharma, Sandoz, and Takeda and as an advisory board member for AbbVie, Ferring, Galapagos, Janssen, and Nestlé. Fernando Magro has served as a speaker for AbbVie, Arena, Biogen, Bristol Myers Squibb, Eli Lilly and Company, Falk, Ferring, Hospira, Janssen, Laboratórios Vitoria, Merck Sharp & Dohme, Pfizer, Sandoz, Takeda, UCB, and Vifor. Shashi Adsul and Christian Agboton are employees of and hold stock/stock options in Takeda.

## Data Availability

Data sharing not applicable to this article as no datasets were generated or analyzed during the current study.
